# Сравнительный анализ экспрессии генов у чайного растения (Camellia sinensis (L.) Kuntze) при низкотемпературном стрессе

**DOI:** 10.18699/VJ20.653

**Published:** 2020-10

**Authors:** L.S. Samarina, A.O. Matskiv, N.G. Koninskaya, T.A. Simonyan, V.I. Malyarovskaya, L.S. Malyukova

**Affiliations:** Federal Research Centre the Subtropical Scientific Centre of the Russian Academy of Sciences, Sochi, Russia; Federal Research Centre the Subtropical Scientific Centre of the Russian Academy of Sciences, Sochi, Russia; Federal Research Centre the Subtropical Scientific Centre of the Russian Academy of Sciences, Sochi, Russia; Federal Research Centre the Subtropical Scientific Centre of the Russian Academy of Sciences, Sochi, Russia; Federal Research Centre the Subtropical Scientific Centre of the Russian Academy of Sciences, Sochi, Russia; Federal Research Centre the Subtropical Scientific Centre of the Russian Academy of Sciences, Sochi, Russia

**Keywords:** Camellia sinensis, gene expression, frost tolerance, transcription factors, protein kinases, lipoxygenases, Camellia sinensis, экспрессия генов, морозоустойчивость, транскрипционные факторы, протеинкиназы, липоксигеназы

## Abstract

Низкотемпературный стресс – один из главных факторов, ограничивающих распространение и снижающих урожайность многих субтропических культур, в том числе и чая. Для эффективной селекции чая на устойчивость к морозу необходимо выявить генетические особенности ответа на холод у устойчивых генотипов и найти маркеры для определения доноров устойчивости в коллекциях. В настоящей работе про- веден сравнительный анализ экспрессии 18 генов (ICE1, CBF1, DHN1, DHN2, DHN3, NAC17, NAC26, NAC30, bHLH7, bHLH43, P5CS, WRKY2, LOX1, LOX6, LOX7, SnRK1.1, SnRK1.2, SnRK1.3), вовлеченных в абиотический стрессовый ответ у двух контрастных по устойчивости генотипов чая в условиях холода и мороза. Низкотемператур- ный стресс индуцировали путем помещения растений в холодильные камеры и снижением температуры до 0…+2 °С на семь дней (холодовой стресс) с последующим снижением температуры до –4…–6 °С на пять дней (промораживание). Кондуктометрическим методом измеряли электропроводность тканей листа, в ре- зультате чего были подтверждены различия по признаку устойчивости у двух исследуемых генотипов чая: холодовое воздействие не приводило к изменению электропроводности тканей листа, но после промора- живания этот показатель возрастал в большей степени у неустойчивого генотипа. Методом qRT-PCR анализи- ровали относительный уровень экспрессии генов на фоне референсного гена актина. При индукции стресса показана повышенная экспрессия всех исследуемых генов. У устойчивого генотипа чая выявлен ряд генов, более активно экспрессирующихся по сравнению с неустойчивым генотипом: ICE1, CBF1, DHN2, NAC17, NAC26, bHLH43, WRKY2, P5CS, LOX6, SnRK1.1, SnRK1.3. Эти гены могут быть маркерами устойчивости для поиска доно- ров в коллекциях геноресурсов. Показано, что у устойчивого генотипа чая экспрессия генов холодового от- вета начинается уже на стадии акклиматизации. Для дальнейших исследований комплексной устойчивости растений к низкотемпературному стрессу актуальным является изучение экспрессии этих генов в других органах чайного растения (побегах, корнях) при разной силе низкотемпературного воздействия.

## Abstract (en)

Low-temperature stress is one of the main factors limiting the distribution and reducing the yield of many subtropical crops, including the tea crop. Efficient breeding to develop frost-tolerant cultivars requires a reliable set of genetic markers for identifying resistance donors, and that is why it is necessary to reveal the specific genetic response in frost-tolerant genotypes in comparison with frost- susceptible ones. In this work, we performed a comparative analysis of the expression of 18 tea genes (ICE1, CBF1, DHN1, DHN2, DHN3, NAC17, NAC26, NAC30, bHLH7, bHLH43, P5CS, WRKY2, LOX1, LOX6, LOX7, SnRK1.1, SnRK1.2, SnRK1.3) under cold and frost conditions in two tea genotypes, tolerant and susceptible. Low-temperature stress was induced by placing the potted plants in cold chambers and lowering the temperature to 0…+2 °С for 7 days (cold stress), followed by a decrease in temperature to –4…–6 °С for 5 days (frost stress). Relative electrical conductivity of leaf was measured in response to the stress treatments, and a significant difference in the frost tolerance of the two tea genotypes was confirmed. Cold exposure did not lead to a change in the electrical conductivity of leaf tissue. On the other hand, frost treatment resulted in increased REC in both genotypes and to a greater extent in the susceptible genotype. Increased expression of all the genes was shown during cold and frost. The genes that were strongly expressed in the tolerant tea genotype were revealed: ICE1, CBF1, DHN2, NAC17, NAC26, bHLH43, WRKY2, P5CS, LOX6, SnRK1.1, SnRK1.3. These genes can be proposed as markers for the selection of frost-tolerance donors in tea germplasm collections. Additionally, it was shown that the tolerant genotype is characterized by an earlier response to stress at the stage of cold acclimation. The study of the expression of the identified genes in different organs of tea plants and in different exposures to low temperature is relevant for further investigations.

## Введение

Низкотемпературный стресс, мороз (< 0 °С) и холод (0…+10 °С), ограничивает ареал распространения многих теплолюбивых культур, в том числе и чая. Перестройка метаболизма в ответ на этот абиотический стресс, включающая накопление антиоксидантов и осмолитов для повышения устойчивости, происходит путем запуска сложных сигнальных путей (Dubouzet et al., 2003). Регуляция этих путей осуществляется основными регуляторными генами: COR (cold-responsive). К ним относят гены-регуляторы холодового ответа ICE (inducer of CBF expression), CBF (C-repeat-binding factor) и их мишени: KIN (cold-induced), LTI (low temperature induced) и RD (responsive to dehydration) (Morsy et al., 2005). Продукты этих генов классифицируют на две группы: первая включает гены метаболизма семейств белков, LEA (late embryogenesis abundant proteins), HSP (heat shock proteins), антифриз-белков, белков метаболизма липидов (LOX ), дегидринов (DHN) и осморегуляторов – сахаров, свободных стеролов, раффинозы, глюкозидов, аминокислот; вторая – транскрипционные факторы, регулирующие передачу сигналов и экспрессию генов ответа на холод (Megha et al., 2018).

Различные по устойчивости к этому фактору культуры характеризуются разнообразными механизмами устойчивости, а у древесных культур, таких как чай (Camellia sinensis (L.) Kuntze), ответ на стресс более сложный и комплексный (Hao et al., 2018). Для чайного растения, основная зона произрастания которого – тропические и субтропические регионы, актуальным является создание морозоустойчивых сортов, позволяющих значительно расширить его ареал и увеличить площади возделывания. Северо- Западный Кавказ – один из самых северных регионов промышленного выращивания чая в мире. Доместикация чайного растения в этом регионе длилась около 150 лет, в течение которых плантации распространились из района Озургетти в Грузии (41°55′27″ N, 41°59′24″ E) на север до Майкопа (44°36′40″ N, 40°06′40″ E). Поэтому коллекции гермоплазмы чая на Кавказе могут быть источником генотипов с повышенной морозоустойчивостью.

Ранее было показано, что у чайного растения гены CsICE1 и CsCBF1 – главные COR-гены ответа и адаптации к низкотемпературному стрессу (Wang et al., 2012; Yuan et al., 2013). Были сообщения также, что экспрессия COR-генов регулируется как CBF-опосредованным АБК-независимым путем, так и bZIP-опосредованным AБК-зависимым путем (Ban et al., 2017). Множество транскрипционных факторов (DHN, WRKY, HD-Zip, NAC, bHLH и др.) и генов синтеза метаболитов запускаются в ответ на холод (Yue et al., 2015; Wang et al., 2016a, b; Chen et al., 2018; Cui et al., 2018; Shen et al., 2018; Zhu et al., 2018). Об этих и других генах нами был сделан детальный обзор последних публикаций (Самарина и др., 2019). Цель настоящей работы – провести сравнительный анализ экспрессии генов, вовлеченных в абиотический стрессовый ответ у чая, в условиях холода и мороза у кавказских генотипов, произрастающих в одном из самых северных регионов промышленного выращивания чая в мире.

## Материалы и методы

**Растительный материал и условия эксперимента.** Объектом исследования были трехлетние вегетативно размноженные горшечные растения чая двух контрастных по устойчивости генотипов (коллекции чая Федерального исследовательского центра «Субтропический научный центр Российской академии наук» (ФИЦ СНЦ РАН)): устойчивой к морозу формы А-2016, произрастающей в полевой коллекции Майкопского филиала ФИЦ СНЦ РАН и неустойчивого к морозу сорта Колхида полевой коллекции ФИЦ СНЦ РАН (Сочи) (Туов, Рындин, 2011; Гвасалия, 2015). Горшечные растения (рис. 1) в количестве 10 шт. каждого генотипа выращивали в объеме 2 л бурой кислой лесной почвы (pH_Н_2_О_ = 5.0). До индукции низкотемпературного воздействия растения в течение месяца находились в лабораторных условиях при температуре 20 ± 2 °С с оптимальным режимом полива и освещением лампами дневного света, фотопериодом 16/8 с интенсивностью освещения 3000 лк. После этого у них брали листья для анализа (контрольная группа). Затем эти растения помещали в холодовые камеры. Индукцию стресса проводили воздействием температурой до 0…+2 °С в течение семи дней (холодовой стресс) с последующим снижением температуры до –4…–6 °C на пять дней (промораживание), световой режим сохраняли прежним.

Для лабораторных анализов на всех этапах исследования (контроль, холод, промораживание) использовали третий (для выделения РНК) и четвертый (для физиологических анализов) листья сверху. Для физиологических анализов и выделения РНК каждая из трех повторностей представляла собой смешанную пробу из листьев от трех растений.

**Фенотипирование устойчивости к холоду.** Кондуктометрическим методом определяли относительную электропроводность тканей (%), стабильность клеточных мембран и повреждение тканей листа (%) – с помощью портативного кондуктометра ST300C (Ohaus). Навеску 200 мг свежего листа погружали в 150 мл деионизированной воды, определяли электропроводность сразу после погружения (L0), затем через 2 ч (L1), далее кипятили на водяной бане 60 мин при 100 °C и определяли электропроводность после остывания раствора (L2). Относительную электропроводность рассчитывали по формуле:

REC (%) = L0/L1 × 100.

Стабильность клеточных мембран (CMI, %) вычисляли по формуле:

CMI = (1 – (L1/L2)) / (1 – (C1/C2)) × 100,

где C1 и C2 – средняя электропроводность контроля до и после кипячения соответственно (Bajji et al., 2002). Степень повреждения тканей оценивали как 100 – CMI. Статистическую обработку полученных данных выполняли методом однофакторного дисперсионного анализа.

Анализ экспрессии генов. Выделение РНК из свежих листьев проводили с использованием наборов реагентов Лира (http://biolabmix.ru/). Качество РНК оценивали методом электрофореза в агарозном геле, ее концентрацию определяли на приборе BioDrop μLite (Serva). Разведенную РНК обрабатывали ДНКазой и подтверждали отсутствие примесей геномной ДНК методом qRT-PCR. Обратную транскрипцию осуществляли набором реагентов M-MuLV-RH (http://biolabmix.ru/). Количественный анализ экспрессии генов методом ПЦР в реальном времени делали на приборе LightCycler96 (Roche). Смесь ПЦР готовили на основе наборов реагентов БиоМастер HS-qPCR SYBR Blue(2×) (http://biolabmix.ru/). Объем смеси – 12.5 мкл, в нее входили по 0.5 мкл каждого праймера (из раствора 10 ммоль) (см. таблицу), 6.25 мкл буфера, 1 мкл кДНК (500 нг/мкл) и вода. Использовали стандартные условия для ПЦР с двухшаговой амплификацией (35 циклов), температурой отжига праймеров 60 °C. В качестве референсного гена был Actin, анализ экспрессии выполняли в трех биологических повторностях, данные обрабатывали с помощью программного обеспечения LightCycler96. Относительный уровень экспрессии гена рассчитывали по алгоритму:

2^–ΔΔCq^,

где ΔΔCq = (Cq_gene of interest_ – Cq _internal control_)_treatment_ – – (Cq_ gene of interest_ – Cq _internal control_)_control_

**Table 1.**
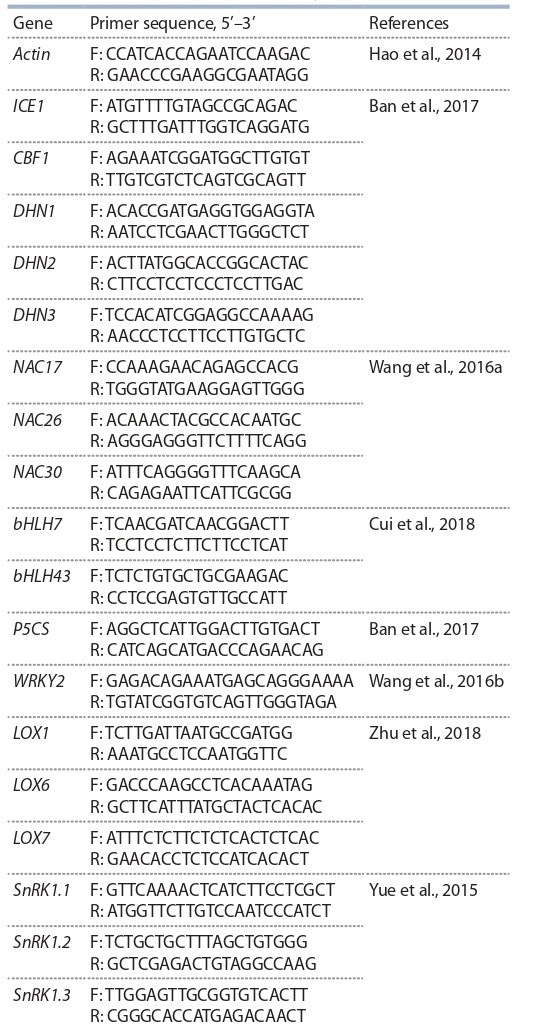
Genes and primers included in the study

## Результаты

Исследования показали, что холодовое воздействие (0…+2 °C) не приводило к существенным изменениям показателей электропроводности тканей листа. При промораживании (–4…–6 °C) значительно возрастал относительный уровень электропроводности, а стабильность клеточных мембран снижалась, что свидетельствует о повышении выхода электролитов из тканей. При этом растения более устойчивого генотипа в меньшей степени повреждались

**Fig. 2.**
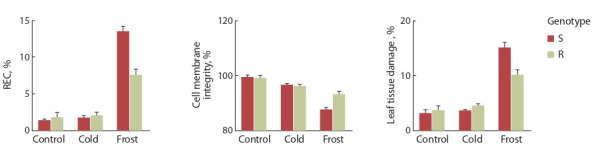
Relative electrical conductivity (REC), cell membranes integrity, tissue damage rate of tea leaves during cold (0…+2 °C) and frost (–4…–6 °C) treatment: S, susceptible genotype; R, resistant genotype.

Относительный уровень экспрессии всех генов, включенных в эксперимент, повышался при индукции низкотемпературного стресса (рис. 3). При сравнении ответа холод/мороз достоверные различия наблюдались по экспрессии генов ICE1, WRKY2, DHN, NAC, bHLH7, LOX1, LOX6, P5CS. По другим генам различия в ответе холод/ мороз несущественны.

**Fig. 3.**
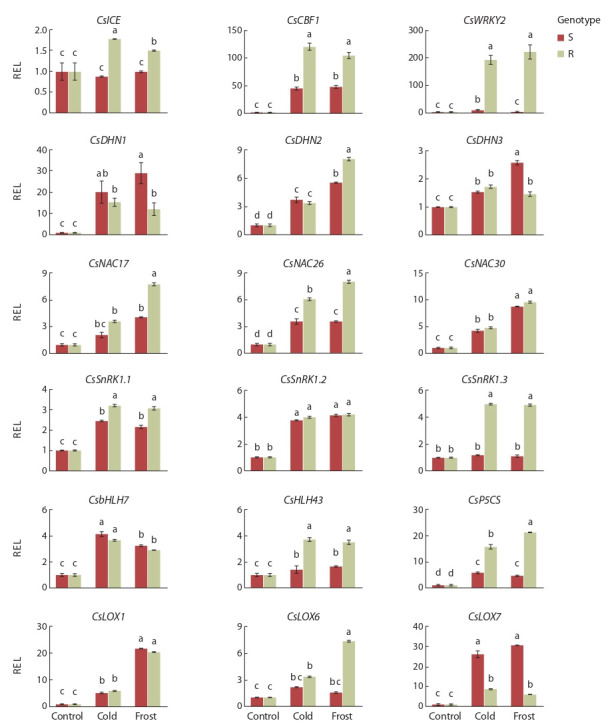
Relative expression level (REL) of cold stress genes in tea leaves (S, susceptible genotype; R, resistant genotype) exposed to cold (0…+2 °C) and frost (–4…–6 °C). Letters above bars indicate significance of differences at p < 0.05.

При сравнении ответа устойчивый/неустойчивый генотип по большинству генов выявлены значимые различия. Из 18 генов 11 проявили более высокий уровень экспрессии у устойчивого генотипа.

Экспрессия гена-регулятора холодового ответа ICE1 у устойчивого генотипа достоверно повышалась в 1.5–1.8 раза при индукции стресса. У неустойчивого генотипа она оставалась на уровне контроля. Транскрипты CBF1 накапливались у устойчивого генотипа в 3 раза сильнее, чем у неустойчивого, и составили 120 и 45 единиц соответственно. Апрегуляция гена WRKY2 усиливалась при холоде и промораживании в большей степени у устойчивого генотипа – в 200 раз. Транскрипты генов дегидринов DHN усиленно накапливались у обоих генотипов при низкотемпературном стрессе. Достоверные различия между генотипами отмечены только по экспрессии DHN2. Оверэкспрессия трех генов семейства NAC наблюдалась при низкотемпературном стрессе у чая, которая в среднем составила 2–9 единиц. При этом устойчивый генотип отличался более высокой экспрессией генов NAC26 и NAC17 в сравнении с неустойчивым. Гены HLH7 и HLH43 также усиленно экпрессировались при холоде и промораживании в 3–4 раза, при этом HLH43 активнее проявился у устойчивого генотипа. По гену HLH7 различий между двумя генотипами не отмечено.

Экспрессия генов семейства LOX также повышалась в 5–26 раз при индукции холода и мороза. У устойчивого генотипа более активно проявился ген LOX6 – в 3 и 7 раз – при холоде и морозе соответственно. Ген LOX7 в большей степени активен при холоде у неустойчивого генотипа, а ген LOX1 экспрессировался одинаково у двух генотипов. Три гена семейства SnRK апрегулировались у обоих сортов, ~ 2–4 раз при холоде и морозе, при этом у устойчивого генотипа отмечен более высокий уровень экспрессии SnRK1.1 и SnRK1.3, по сравнению с неустойчивым сортом.

В целом сравнительный анализ экспрессии генов у двух генотипов показал, что устойчивый генотип раньше реагирует на стресс, транскрипты ряда генов у него сильнее накапливаются уже на этапе холодовой акклиматизации. Гены ICE1, CBF1, WRKY2, DHN2, NAC17, NAC26, SnRK1.1, SnRK1.3, bHLH43, P5CS, LOX6, проявились в большей степени у устойчивого генотипа чая при низкотемпературном воздействии и могут быть маркерами для отбора доноров устойчивости к холоду

## Обсуждение

Проведен сравнительный анализ экспрессии генов у двух контрастных по устойчивости генотипов чая в условиях индукции холода и промораживания для выявления различий в их ответных реакциях.

В анализ был включен ряд генов, которые, как ранее сообщалось, играют или могут играть важную роль в ответе на холодовой стресс. В опубликованных транскриптомных исследованиях показано, что ответ на холод и мороз у чайного растения различается (Li et al., 2019), поэтому мы сравнивали относительный уровень экспрессии генов в условиях этих двух стрессовых воздействий. В результате обнаружен ряд генов, которые экспрессируются по- разному в условиях холода и мороза; установлены гены, различающиеся по уровню экспрессии у устойчивого и восприимчивого к морозу генотипов. Сравнение уровня экспрессии у двух различных по устойчивости генотипов может помочь предположить, какие из этих генов могут быть маркерами для поиска доноров устойчивости в селекции чая.

Низкотемпературный стресс приводит к нарушению стабильности клеточных мембран, изменению липидного, белкового и ферментного баланса клетки, вызванному окислительными процессами (Somerville, 1995; Thomashow et al., 1999). Наши результаты подтвердили, что А-2016 более морозоустойчив, чем сорт Колхида, так как у него в большей степени сохранялась целостность клеточных мембран при индукции заморозков.

Обнаружено, что у устойчивого генотипа более активно экспрессировались гены ICE1, CBF1, DHN2, NAC17, NAC26, bHLH43, WRKY2, P5CS, LOX6, SnRK1.1, SnRK1.3 и их экспрессия была существенно выше уже на стадии холодовой акклиматизации, т. е. на первом этапе индукции стресса. Эти результаты согласуются с данными опубликованных исследований, в которых повышенная устойчивость растений чая обусловлена более скорым ответом на холодовой стресс (Ban et al., 2017; Li et al., 2019).

Известно, что DHNs – группа генов, кодирующих белки- дегидрины, которые действуют как криопротекторы, молекулярные шапероны, а также антиоксиданты, играя базовую роль в ответе растений на абиотические стрессы, входят в семейство транскрипционных факторов LEA II (Late Embryogenesis Abundant) (Hanin et al., 2011). Из трех генов DHN, ген DHN2 может служить маркером холодоустойчивых сортов чая, что согласуется с ранее опубликованными данными по китайским генотипам чая (Ban et al., 2017).

Семейство транскрипционных факторов NAC кодирует белки, играющие важную роль в передаче ауксинового сигнала и развитии меристем, латеральных корней, клеточной стенки, биосинтезе флавоноидов и др. По данным других исследователей, экспрессия этих генов индуцируется засухой, засолением, холодом и повышением содержания абсцизовой кислоты (АБК) (Wang et al., 2016a). Наши результаты также подтверждают повышение уровня экспрессии этих генов у обоих генотипов как при холоде, так и при промораживании.

Гены семейства WRKY участвуют в АБК-зависимом пути ответа на абиотический стресс, а также в регуляции роста и развития растений. У чайного растения был выделен новый ген этого семейства, CsWRKY2, экспрессия которого повышалась при холодовом стрессе (Wang et al., 2016b). Полученные нами результаты согласуются с этими данными, к тому же у устойчивого генотипа CsWRKY2 проявился более активно и при холоде, и при морозе, поэтому мы предполагаем, что он также может быть одним из маркеров устойчивости.

Гены SnRK1.1, SnRK1.2, SnRK1.3 кодируют ферменты протеинкиназы, регулирующие катаболизм углеводов, экспрессию генов метаболизма сахарозы, в частности генов SUS. В ответ на холод у чайного растения экспрессия этих генов существенно возрастала, что отмечено и у китайских сортов чая (Yue et al., 2015). Кроме того, по нашим данным, устойчивый генотип характеризовался более активной экспрессией SnRK1.1 и SnRK1.3, следовательно, углеводный метаболизм у него протекал более активно.

К семейству транскрипционных факторов bHLH, участвующих в широком спектре биологических процессов, относятся: вторичный метаболизм брассиностероидов, жасмоновой кислоты, синтез антоцианов, модуляция роста и развития растений, контроль ветвления побегов и др. Кроме того, bHLH играют важную роль в передаче сигнала АБК и ответе растений на абиотические стрессы. У чайного растения обнаружено 39 генов CsbHLH, экспрессия которых повышалась в условиях засухи (Cui et al., 2018). Мы анализировали экспрессию двух генов из этого семейства, и оба из них апрегулировались при низкотемпературном стрессе, а ген bHLH43 активнее экспрессировался у устойчивого генотипа.

Гены семейства липоксигеназ LOX вовлечены в катаболизм липидов, синтез оксилипина, жасмоновой кислоты и C6-альдегидов (Li et al., 2017). У чайного растения обнаружено, что гены CsLOX1, CsLOX6 и CsLOX7 могут играть важную роль в ответе на стрессы (холод, засуху, биотический стресс) в АБК-независимом пути ответа (Zhu et al., 2018), поэтому мы включили эти гены в эксперимент. В наших результатах показано, что эти три гена экспрессировались сильнее при холоде и промораживании, в сравнении с контролем, однако у устойчивого генотипа только ген LOX6 более активно экспрессировался при стрессе, в сравнении с неустойчивым.

Ген P5CS – один из генов, вовлеченных в синтез пролина (Szekely et al., 2008). Более высокий уровень его экспрессии наблюдался при холоде у устойчивого генотипа чая. Достоверных различий экспрессии этого гена между устойчивыми и неустойчивыми сортами чая не отмечено (Ban et al., 2017). Проведение дополнительных исследований с разными сортами поможет верифицировать полученные данные.

## Заключение

Таким образом, показана повышенная экспрессия всех изучаемых генов: DHN1, DHN2, DHN3, NAC17, NAC26, NAC30, bHLH7, bHLH43, WRKY2, LOX1, LOX6, LOX7, SnRK1.1, SnRK1.2, SnRK1.3. Выявлен ряд генов, более активно экспрессирующихся у устойчивого генотипа чая: ICE1, CBF1, DHN2, NAC17, NAC26, bHLH43, WRKY2, P5CS, LOX6, SnRK1.1, SnRK1.3. Обнаружены гены, различающиеся по экспрессии в холоде и в морозе: ICE1, WRKY2, DHN, NAC, bHLH7, LOX1, LOX6, P5CS. В целом устойчивый генотип характеризуется более ранним ответом на стресс. Однако в нашей работе проанализировано только два генотипа чая, поэтому для дальнейшей верификации маркеров устойчивости к холоду необходимо привлечение большего количества генотипов. Для дальнейших исследований актуально изучение экспрессии этих генов в других органах растений чая при разной силе низкотемпературного воздействия.

## Conflict of interest

The authors declare no conflict of interest.
